# Differences among Three Measures of Reaction Time Based on Hand Laterality in Individual Sports

**DOI:** 10.3390/sports6020045

**Published:** 2018-05-19

**Authors:** Dana Badau, Bilgehan Baydil, Adela Badau

**Affiliations:** 1 Department of Human Movement Sciences, University of Medicine and Pharmacy, 540139 Tirgu Mures, Romania; adela.badau@umftgm.ro; 2 Interdisciplinar Doctoral School, Transilvania University of Brasov, 500036 Brasov, Romania; 3 Department of Physical Education and Sports, Kastamonu University, 37200 Kastamonu, Turkey; bilgehan@kastamonu.edu.tr; 4 Department of Physical Education, University of Medicine and Pharmacy, 540139 Tirgu Mures, Romania; adela.badau@umftgm.ro

**Keywords:** reaction time, visual stimulus, hand laterality, individual sports, computer games

## Abstract

(1) Aim: The study aimed at assessing simple-reaction, recognition and cognitive-reaction times to visual stimuli among student athletes: boxing, gymnastics, taekwondo, judo, karate and wrestling, using computer games tests. (2) Methods: Our study involved 332 students and athletes. We applied three types of computer tests to measure the dominant and non-dominant hands: the simple motor reaction time through the Human Benchmark test, the recognition time by the Hit-the-dots and the cognitive reaction time by the Trail making test part B. (3) Results: For dominant and non-dominant hands, better results of individual sports were for: simply reaction time—boxing; recognition reaction time—taekwondo; cognitive reaction—judo. (4) Conclusions: Athletes had better simple reaction with the left hand than with the right hand. Athletes had better recognition and cognitive reaction time with the right hand than with the left hand regardless of the dominant hand. The outcomes of our study indicate that the reaction times of left and right hands were influenced by the hand laterality, the type of applied stimulus, the stress complexity of tests and the type of practiced sport.

## 1. Introduction

Simple reaction time (SRT) is a simple reaction to a single stimulus. Recognition reaction time (RRT) involves a cognitive process of selecting the optimal response to multiple stimuli, and the response is dependent on the type and form of the stimuli. Cognitive reaction time (CRT) consists of identifying the significance of the stimuli, the association and application of knowledge in order to develop an optimal cognitive response in agreement with the stimulus complexity. Research on the hand laterality facilitated the understanding of the way of training and control: characteristics and reaction time of motor skills, responsiveness and motor coordination, thus contributing to optimizing human motor capacity. 

Factors that can affect the average human reaction time (RT) include: age, sex, left- or right-hand dominance, central and peripheral vision, practice, fatigue, fasting, breathing cycle, types of personality, exercise and intelligence of the subject [1]. 

Reaction time components are: mental processing time, afferent nerve conduction time, movement analysis time and device response time [2,3], and they are affected by: age [4], training condition [2,5, neuromuscular fatigue level [6,7], biological rhythm and, health conditions [3,8].

Computer-based assessment of reaction time offers benefits over paper-and-pencil measures in the form of millisecond timing accuracy, reliable and randomized presentation of stimuli over multiple trials and repeated administrations and unobtrusive measurement of cognitive skills and reaction times during all aspects of the assessment process [9]. Computer games are used in physical education and sport in order to improve some skills and optimize the evaluation process [3,10,11].

An innovative aspect of this study consists highlighting the interrelationships between the dominant and non-dominant hand (hand-D and hand-nonD), as well as the specific executions with the right and the left hand of the athletes in six studied sports in terms of SRT, RRT, CRT. 

The aim of the study was to explore the differences between simple reaction time, recognition reaction time and cognitive reaction time, between the right hand and the left hand according to the types of sport using computer games as measurement tools. The results of the study should enhance our understanding of the interrelationship between the dominant and non-dominant hand actions in optimizing sports performances.

## 2. Materials and Methods

### 2.1. Study Design

The research was conducted between March 2016 and June 2017. The dominant hand was established by asking the subjects which hand they used while performing various motor actions. The students were asked about the hand used on the computer mouse. Within the study, we applied three types of measurement tests: the simple motor reaction time (SRT) through the Human Benchmark [12], the recognition time (RRT) by means of the Hit-the-dots [13] and the cognitive reaction time (CRT) by using Trail Making Test part B (TMT). The tests were administered in two sessions with 30-min breaks between sessions. After the informed consent, the tests were administered under the supervision of the authors. This study was approved by the ethics committee of the Transilvania University of Brasov. Participation in the study was voluntary and informed written consent was taken from every participant and the study follows the principles of the Declaration of Helsinki.

### 2.2. Participants

The study aimed at assessing SRT, RRT and CRT. This cross-sectional study included 332 students specialized in physical education and sports: 187 (56.32%) from the University of Medicine and Pharmacy Targu Mures, Romania and 145 (43.68%) from Kostamonu University, Turkey. The inclusion criteria were: active athletes, participants in national and international competitions in 2016 and 2017. The subjects were randomly selected for the study. The exclusion criteria: practicing other types of individual sports than those under study; upper body injuries requiring more than one month of rest. The subjects of the study were active athletes practicing various sports at a competitive youth and senior levels individual sports practitioners (ISG). The distribution of athletes according to the practiced sport by ISG included: boxing 46 (13.9%), gymnastics 58 (17.5%), judo 64 (19.3%), karate 62 (18.7%), wrestling 48 (14.4%), taekwondo 54 (16.2%). The ISG included 278 right-handed laterality athletes (83.7%) and 54 left-handed laterality athletes (16.3%). Age characteristics of the mean age sample ± SD 20.38 ± 1.20; right handed group 20.21 ± 1.20; left handed group 20.11 ± 1.07; male group 20.42 ± 1.173; female group 19.83 ± 1.136. 

### 2.3. Measures

SRT, with the computer-gaming reaction test of Human Benchmark, was designed to evaluate the reaction time to frontal visual stimuli. The testing was carried out with dominant and non-dominant hands, five executions for each hand/2 trials and we took into consideration the average of reaction time for the best trial result in miliseconds (ms). Instructions: when the red box turns green, click as quickly as you can; five trials for each hand, counting the best average in seconds from two sessions/hand-D and hand-nonD. RRT with Hit-the-dots Reaction Test designed by University of Washington [13]. The goal of this computer game consists of finding as many black dots and hitting them in 30 s. The test comprised 60 white small circles with a black border, arranged in 6 lines of 10 circles. Instructions: Click on the black dots as they appear in the white circles; counting how many dots you clicked in 30 s, 1 point per hit, minus 1 point per miss; do the test twice. CRT was measured with the Trail Making Test (TMT) part B [14,15]. The TMT-B is used as a measure for cognitive flexibility [16]. The TNT part B of the test include a number of 25 circles randomly arranged on the phone display, the circles are combined with letters, the numbers are from 1 to 13 and the letters from A to L. The subjects had to identify and associate the figures with the letters in an ascending order by clicking on 1-A model; 2-B, 3-C etc. up to 13. 

### 2.4. Experimental Protocol

The participants of the study were asked to perform as quickly as possible. Data from the best of two trials for both hands were used for analysis. Subjects were allowed a trial test before starting each test session. The tests were applied in different evaluation sessions with a 30 min break. The order of the tests was: SRT, RRT, CRT. The subjects were initially tested with the hand-D and then with the hand non-D. For the Benchmark test and the Hit-the-dot test we used Dell computers with CORE I7 processor of the same generation, the Hewlett-Packard mice were identical, and for the TMT test, we downloaded the application to Iphone 6s phones.

### 2.5. Statistical Analysis

The data were processed using IBM-SPSS 20. The main statistical indicators were: mean, standard deviation (SD), mean difference (MD), partial effect size (ηp2). Descriptive statistics (mean ± SD) were calculated for all variables. The value of statistical significance was set at *p* < 0.05. For parametric comparison of two groups we used *t*-test. For repeated measures comparisons we have used General Linear Model with the dominant arm variable as between the subjects factor. 

## 3. Results

Our study revealed that all subjects normally use the right hands. However, some of them stated that the left hands were dominant.

In individual sports, the differences were statistically significant. *p*_value_ < 0.045, so the athletes with the right hand-D, at Benchmark test, hand-D had lower SRT performance than hand-nonD being slower by 4.75 ms. SRT to visual stimuli the differences being insignificant as follows: between right hand-D compart with left hand-nonD 8.37 ms; between left hand-D and right hand-nonD −13.91 ms; between right-left hands-D 9.03 ms; between right-left hands-nonD −13.25 ms. For ISG hand-D they perform faster than hand-nonD by 7.39 ms. 

RRT measured with Hit-the-dot test (Table 1) had better results to the right hand compared to the left hand, regardless of the dominant hand, the difference was statistically significant. *p*_value_ < 0.001. RRT for ISG showed a statistically superior and significant reaction of hand-D compared with the hand-nonD by 7.81 points, *p*_value_ 0.025. RRT to visual stimuli revealed the following differences: between right hand-D compart with left hand-nonD 10.61 points; between left hand-D and right hand-nonD −6.57 points; between right-left hands-D 9.68 points; between right-left hands-nonD −7.50 points. 

In the TMT test (Table 1), athletes had better cognitive reactions with the right hand than with the left hand regardless of the hand-D. For TMT-B, the CRT difference revealed the following differences: between hand-D right compart with left hand-nonD −1.37 s; between left hand-D and right hand-nonD 6.08 s; between right-left hands-D −3.08 s; between right-left hands-nonD 4.37 s. For ISG hand-D they perform faster than hand-nonD by 7.39 ms, the difference was no statistically significant. 

In the Benchmark test (Table 2), the differences between hand-D and hand-nonD for individual sports was 7.42 ms. *p*_value_ 0.001. About SRT, except for judo, wrestling had a more marked difference between D-nonD hands intersecting gymnastics, karate and judo. For individual sports at Hit-the-dot test, RRT differences between hand-D and hand-nonD were of 9.90 s, *p*_value_ 0.001. In individual sports, CRT did not record interaction; all sports gave similar results; they were all on the same descending slope and all sports had recorded better times for hand-D versus hand-nonD. TMT-B for CRT, the differences between hands-D and hand-nonD were 0.82 s to individual sports, with no statistical significance. Interaction to TMT-B for CRT individual sports does not show significant differences, most of them have better times at hand-D. A significant difference between D-nonD hands is represented by gymnastics which intersects with wrestling and karate which intersects with judo.

## 4. Discussion

The group distribution of individual sports in terms of hand-D was homogeneous *p*_value_ 0.294. In individual sports, in the Benchmark test, left arm significantly performed better than the right arm, no matter which was dominant. In individual sports, in the Hit-the-dot test, the right hand significantly performed better than the left hand, no matter which was dominant, *p*_value_ < 0.001. 

In individual sports, only in the Hit-the-dot test (η*_p_*^2^ 0.035) differences between hands D-nonD were significant, but the effect size was small. 

In SRT, boxing had a better time, taekwondo had a weaker time, MD ± SD 59.05 ± 7.633, *p*_value_ < 0.000. According to RRT, boxing had better times than taekwondo which had the lowest result, MD ± SD 6.67 ± 1.190, *p*_value_ < 0.000. In CRT, karate had better time and taekwondo weaker time, MD ± SD 7.737 ± 3.10, but without a significant statistical difference. Our results are in accordance with previous studies. One study performed in 2017 on six volunteers who participated in the tests of simply reaction time found that intense sport results in increased reaction times and the light physical activity had a positive effect on human reaction time, resulting in a shorter reaction time in both simple and recognition tests [2]. Although a previous study suggested that left-hand movement simple reaction time might be shorter than that for the right hand [4,17,18,19], another found that the right-hand reaction time was shorter [20,21]. Many studies demonstrated that males had faster SRT compared to females, likely because of the differences in motor responses as opposed to differences in muscle contraction [22,23,24]. Some studies found some statistically significant information though small differences in cognitive abilities between right- and left-handed individuals [17,25]. A study conducted on athletes showed that SRT was significantly better in karate-kumite practitioners than in karate-kata ones. Two-choice RT was also significantly better in karate-kumite practitioners than in karate-kata ones; hockeyball goalies achieved significantly better values than hockeyball players in both simple RT and two-choice RT, soccer goalies surpassed soccer players in both simple RT and two-choice RT [26]. 

The implementation of information technology in the sports field is increasingly complex, aiming at both monitoring the training process and evaluating the motor and functional parameters of athletes.

## 5. Conclusions

For hand-D and hand-nonD, the better results of individual sports were for: simply reaction time—boxing; recognition reaction time—taekwondo; cognitive reaction—judo. In Individual sports for SRT, left arm significantly performed better than the right arm. In individual sports for RRT, right hand significantly performed better than the left hand. Regarding CRT in individuals, the right arm significantly performed better than the left hand, no matter which was dominant. No significant differences were found between right or left hands in the TMT test.

The more complex the application is, the more efficient the dominant hand is. Considering the simple reaction time, recognition and cognition through test computer games makes it possible to get an easy and attractive sports assessment. Future research will extend the research line by applying our results to other sports, on different sport age categories by analyzing the differences between D-nonD hands through other types of tests of computer games.

## Figures and Tables

**Table 1:** Descriptive statistics for the results of the SRT, RRT and CRT for individual sports according with hand laterality.

Tests	Benchmark (ms)			Hit-the-dot (points)			TMT Part B (s)		
Hands (n)	Hand-D	Hand-nonD	*p* *	Hand-D	Hand-nonD	*p* *	Hand-D	Hand-nonD	*p* *
	Mean ± SD	Mean ± SD		Mean ± SD	Mean ± SD		Mean ± SD	Mean ± SD	
Right (278)	253.42 ± 27.11	245.05 ± 32.64	0.045	29.02 ± 4.46	18.41 ± 4.46	0.001	59.14 ± 11.25	60.51 ± 10.87	0.038
Left (54)	244.39 ± 40.49	258.30 ± 31.52		19.34 ± 6.24	25.91 ± 6.15		62.22 ± 9.17	56.14 ± 11.16	
Total (332)	251.95 ± 27.23	247.20 ± 32.21	0.625	27.44 ± 4.76	19.63 ± 4.29	0.025	59.64 ± 11.08	59.79 ± 10.47	0.176

n—number of subjects; * Computed using Repeated Measures of General Linear Model with the hand-D variable as between-subjects factor.

**Table 2:** Statistical analyses of the results of the SRT, RRT and CRT for sports for hand-D and hand non-D.

Tests	Benchmark (ms)			Hit-the-dot (points)			TMT Part B (s)		
Hands	Hand-D	Hand-nonD	* *p*	Hand-D	Hand-nonD	* *p*	Hand-D	Hand-nonD	* *p*
Sports (n)	Mean ± SD	Mean ± SD		Mean ± SD	Mean ± SD		Mean ± SD	Mean ± SD	
Boxing (46)	222.78 ± 19.51	215.83 ± 20.24	0.023	33.78 ± 5.05	21.28 ± 5.09	0.249	61.32 ± 4.74	62.61 ± 5.00	0.119
Gimnastics (58)	256.55 ± 22.45	252.17 ± 29.85		28.17 ± 4.77	19.97 ± 4.87		57.22 ± 13.35	61.96 ± 17.38	
Judo (64)	243.78 ± 29.97	248.34 ± 24.49		27.16 ± 5.38	17.78 ± 3.96		57.30 ± 6.18	57.71 ± 7.46	
Karate (62)	255.65 ± 20.26	241.27 ± 36.14		29.04 ± 3.80	19.69 ± 4.84		56.83 ± 12.35	58.13 ± 10.37	
Taekwondo (54)	280.71 ± 22.93	276.00 ± 22.75		26.00 ± 3.57	15.71 ± 3.17		65.82 ± 7.41	64.61 ± 5.78	
Wrestling (48)	261.25 ± 21.14	239.67 ± 35.04		28.17 ± 4.09	17.67 ± 5.15		60.57 ± 15.54	58.45 ± 10.18	
Total (332)	253.85 ± 27.43	246.42 ± 32.32	0.001	28.56 ± 4.93	18.65 ± 4.47	<0.001	59.61 ± 10.04	60.43 ± 9.52	0.343

n—number of subjects; * Computed using Repeated Measures of General Linear Model with the dominant arm variable as between-subjects factor.
